# Soliton superlattices in twisted hexagonal boron nitride

**DOI:** 10.1038/s41467-019-12327-x

**Published:** 2019-09-25

**Authors:** G. X. Ni, H. Wang, B.-Y. Jiang, L. X. Chen, Y. Du, Z. Y. Sun, M. D. Goldflam, A. J. Frenzel, X. M. Xie, M. M. Fogler, D. N. Basov

**Affiliations:** 1 Department of Physics, Columbia University, New York, NY 10027 USA; 2 State Key Laboratory of Functional Materials for Informatics, Shanghai Institute of Microsystem and Information Technology, Chinese Academy of Sciences, 865 Changning Road, 200050 Shanghai, P. R. China; 3 Department of Physics, University of California, San Diego, La Jolla, CA 92093 USA

**Keywords:** Two-dimensional materials, Polaritons

## Abstract

Properties of atomic van der Waals heterostructures are profoundly influenced by interlayer coupling, which critically depends on stacking of the proximal layers. Rotational misalignment or lattice mismatch of the layers gives rise to a periodic modulation of the stacking, the moiré superlattice. Provided the superlattice period extends over many unit cells, the coupled layers undergo lattice relaxation, leading to the concentration of strain at line defects – solitons - separating large area commensurate domains. We visualize such long-range periodic superstructures in thin crystals of hexagonal boron nitride using atomic-force microscopy and nano-infrared spectroscopy. The solitons form sub-surface hexagonal networks with periods of a few hundred nanometers. We analyze the topography and infrared contrast of these networks to obtain spatial distribution of local strain and its effect on the infrared-active phonons of hBN.

## Abstract (web summary)

Solitons may develop when strain forms at line defects separating commensurate domains in misaligned or lattice-mismatched van der Waals heterostructures. Here, the authors use atomic-force microscopy and nano-infrared spectroscopy to image solitons in thin hBN crystals in the form of long-range periodic superstructures, creating sub-surface hexagonal networks with periods of a few hundred nanometers.

## Introduction

Periodic modulation of interlayer stacking in two-dimensional van der Waals (vdWs)-based systems provide a unique control of their physical properties^1–18^ not available in commonplace epitaxial heterostructures. Prominent examples include twisted bilayer graphene (TBG), graphene on lattice-mismatched hBN substrate^1,3–8,10–18^, and hetero-bilayers of transition metal dichalcogenides^9,19^. In particular, the moiré superlattice in TBG at the “magic” twist angle *θ* ≈ 1° gives rise to strong electron correlations and superconductivity^2,20^. On the other hand, a network of solitons forming in TBG at smaller *θ*^2,11,13,14,21,22^, radically alters its electronic^13,21–24^ and plasmonic^14,23,25^ properties. Here we report on nano-imaging of soliton superlattices in hexagonal boron nitride (hBN). The solitons appear to originate at a misfit atomic plane located ~15 nm beneath the surface of hBN crystals. Utilizing scattering-type scanning near-field optical microscope (s-SNOM), we uncovered that networks of these buried soliton superlattices are registered in infrared (IR) spectral features associated with dipole-active phonons of hBN. We modeled the near-field IR contrast of the solitons in terms of local hardening and broadening of the phonon modes, which we related to the distribution of the elastic strain in the system.

## Results

### Topography of hBN domain patterns

**Fig. 1:**
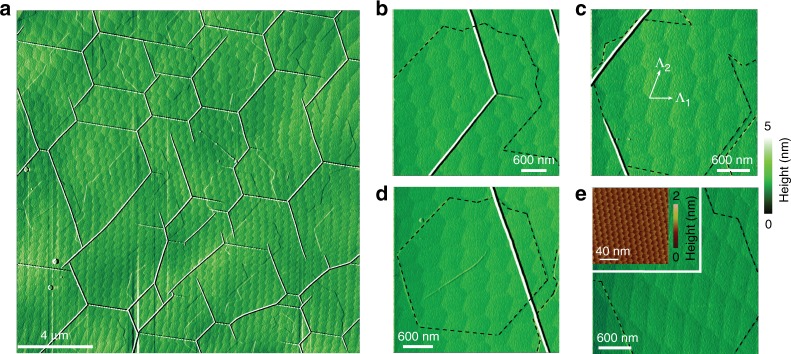
AFM topography images. **a** Large-scale image of hBN crystals showing the periodic domain pattern. **b**–**e** Zoom-in view of different smaller regions revealing varied domain shapes. The dashed black lines are the contours of graphene epitaxial grown on hBN. Inset of **e** displays a high-resolution topography (friction-AFM) image demonstrating the small-period Moiré pattern at the graphene/hBN interface. The large-period domain patterns are influenced neither by the presence of graphene nor by hBN wrinkles (the white lines in **a**–**d**) on the top surface of the crystal

We begin with the topographic images of the hBN domain patterns (Fig. 1a–e) obtained with the atomic force microscope (AFM). The patterns extend over macroscopic areas (**>**10^4^ μm^2^) and have periods Λ_1_, Λ_2_ ~ 300–800 nm. The domains vary from nearly perfectly hexagonal (Fig. 1b) to highly distorted, diamond-like motifs (Fig. 1e). The domain boundaries are demarcated by the dips in the topography, which are about *w* ~ 90 nm wide (Fig. S2 of Supplementary Information). Macroscopic domain patterns are not impacted by defects on the sample surface such as wrinkles^26^: the white lines of elevated topography in Fig. 1a–d. Similarly, the domain boundaries do not disturb the 14-nm-periodic moiré pattern formed by monolayer graphene deposited on top of hBN (Fig. 1e, inset). Both observations suggest that the long-range superlattices form at some depth *d* below the surface of the hBN crystal. Based on a theoretical model for the strain distribution around a soliton, we estimate *d* ~ *w*/7 ~ 15 nm (Supplementary Information Section 2).

### Origin of hBN soliton superlattices

**Fig. 2:**
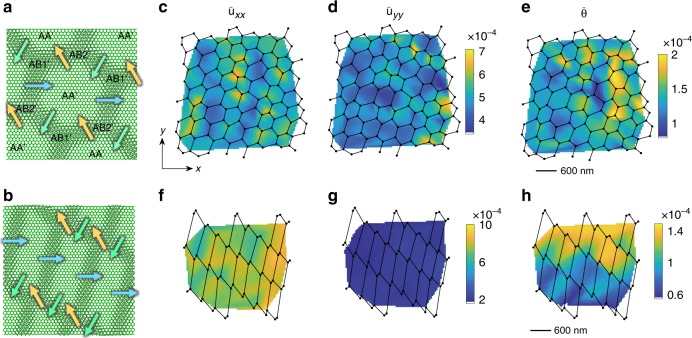
**a**, **b** Schematic diagram of the soliton superlattice in hBN. The shift of the lattice sites in the two adjacent misfit atomic layers (green and gray) is concentrated at the solitons (misfit dislocations). Each soliton is characterized by one of the three possible Burgers vectors displayed with arrows. The regions of AA’, AB1’, and AB2’ stacking are labeled. **c**–**h** Solitons (lines), their junctions (dots), and the corresponding maps of coarse-grained quantities: **c**, **f** *x*-axis average tensile strain $\bar u_{xx}$, **d**, **g** *y*-axis average tensile strain $\bar u_{yy}$, **e**, **h** average rotation angle $\bar \theta$ (rad). The average shear strain $\bar u_{xy}$ is small everywhere and not shown. The top row panels **c**–**e** are deduced from Fig. 1b; the bottom row panels **f**–**h** are obtained from Fig. 1e

For an in-depth look at the solitons, we focus on two representative cases (Fig. 1b, e). The locations of the domain-wall junctions in these images, connected by straight lines, are plotted in Fig. 2c–h. These latter plots reveal somewhat deformed lattices whose primitive periods Λ_1_, Λ_2_ (Fig. 1c) vary in space. We can relate such variations to the interlayer strain and rotation in the system, or more precisely, to the averaged (coarse-grained) strain tensor $\bar u_{\alpha \beta }$ and twist angle $\bar \theta$. Figure 2 illustrates the calculated color maps of $\bar u_{\alpha \beta }({\mathbf{r}})$ and $\bar \theta \left( {\mathbf{r}} \right)$ superimposed on the soliton meshes. Here *α*, *β* ∈ {*x*, *y*} and **r** = (*x*, *y*) is the in-plane position. Note that the angles at all the soliton junctions are close to 120°, which is analogous to the Plateau law of foam films^27^ (see Supplementary Information Section 3 for details). For domains shaped as unilateral hexagons, we find an isotropic and predominantly tensile average strain $\bar u_{xx},\bar u_{yy} \sim 0.05\%$ with the average twist angle $\bar \theta \sim 10^{ - 4}\,{\mathrm{rad}} \approx 0.01^\circ$. For diamond-like domains, the strain is anisotropic, almost uniaxial. A couple of remarks on these results are in order. First, the coarse-grained strain $\bar u_{\alpha \beta }$ should not be confused with the local strain *u*_*αβ*_, which is to be discussed below. Second, the calculation of $\bar u_{\alpha \beta }$ and $\bar \theta$ requires as an input the Burgers vector **b**_*j*_ of the solitons. These vectors connect the nearest identical atoms (e.g., borons) in the same atomic plane. They have magnitude *b* = 0.25 nm each and angular directions separated by 120°. However, since our AFM does not resolve the hBN crystal orientation, we do not know these directions. To generate Fig. 2 we chose one of these directions along the *x*-axis of the plot (Fig. 2a, b). If the Burgers vectors are rotated through some common angle, qualitatively similar $\bar u_{\alpha \beta }({\mathbf{r}})$ and $\bar \theta \left( {\mathbf{r}} \right)$ maps are obtained.

### Nano-IR imaging of hBN superlattices

**Fig. 3:**
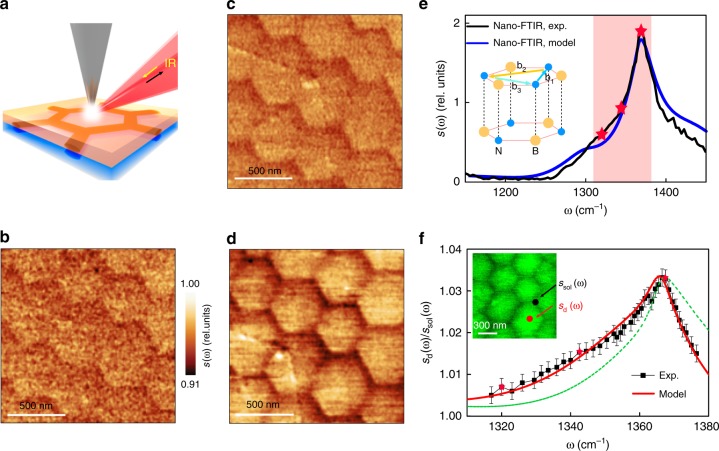
Solitons and lattice dynamics of hBN by nano-IR imaging and spectroscopy. **a** Schematic of the nano-IR imaging showing an AFM tip illuminated by a focused IR beam. The solitons reside at the atomic interface between two parts of the hBN slab (yellow and blue regions). The orange and blue cylinders delineating one hexagonal domain inside the slab represent the strain distribution around the solitons. **b**–**d** Nano-IR images of hBN domain patterns at frequencies 1320, 1344, and 1368 cm^−1^, respectively. These frequencies are marked with red stars and dots in panels e and f. **e** Typical nano-FTIR spectrum of an hBN crystal. Black line: experimental data, blue spectrum: theoretical model described in the text. The inset illustrates the AA′ stacking of hBN and the three possible Burgers vectors **b**_*j*_. The shaded area highlights the spectral range analyzed in panel **f**. **f** The *s*_d_(*ω*)/*s*_sol_(*ω*) spectra across the hBN phonon polariton band. The error bars represent the 90% confidence intervals. The red solid line is the best fit to the data (black squares) using the frequency and the damping rate of the hBN optical phonon as adjustable parameters. The green line is the best fit obtained by varying the damping rate only. The inset shows the AFM topography corresponding to the IR images in panels **b**–**d**

We proceed to the survey of the results obtained by the IR nano-imaging. We have carried out these scanning nano-spectroscopy experiments in the frequency region of hBN phonon polaritons^28–32^. In our s-SNOM apparatus the metalized AFM tip was illuminated by IR light thus generating a strong enhancement of the electric field underneath the tip (Fig. 3a)^4,33^. Such an antenna-based nano-IR setup solves the problem of the photon-polariton momentum mismatch^33–35^ and enables local spectroscopy of polariton modes in hBN with ~25 nm spatial resolution (see the “Methods” section). Employing tunable quantum cascade lasers, we acquired nano-IR images at more than 50 different discrete frequencies. We found that the domain patterns revealed by the AFM topography are also prominent in the nano-IR images (Fig. 3b–d). We obtained nano-IR data with both monochromatic tunable quantum cascade lasers (Fig. 3b–d, f) and also broadband difference frequency generation sources (Fig. 3e) using Fourier transform spectroscopy. The strongest contrast is observed at frequency *ω* = 1368 cm^−1^ (Fig. 3d), which corresponds to the phonon polariton resonance of hBN (Fig. 3e). The contrast systematically weakens as *ω* is shifted away from *ω* = 1368 cm^−1^. In all the images the back-scattering amplitude *s*(*ω*, **r**) is enhanced at the centers of the domains (*s* = *s*_d_(*ω*)) and reduced at the solitons (*s* = *s*_sol_(*ω*)). We therefore conclude that the lattice dynamics is modified near the solitons where the strain is concentrated. The frequency dependence of the ratio *s*_d_(*ω*)/*s*_sol_(*ω*) highlights subtle yet systematic variations of this modified lattice response. This ratio exhibits an asymmetric maximum, with a broader low-frequency side (Fig. 3f).

### Elucidating nano-IR response

Quantitative modeling of the nano-IR response is a challenging task that goes beyond the scope of the present work. To analyze the *s*_d_(*ω*)/*s*_sol_(*ω*) spectra, we restricted ourselves to the simplified approach, similar those in recent nano-IR study of wrinkled and strained hBN crystals^26^ and an earlier work on nano-indented SiC^36^. We assumed that the hBN crystal can be described by a permittivity tensor *ε*_*α*_(*ω*, **r**) varying only as a function of *in-plane* coordinates. Here *α* = ⊥(||) is the in-plane (out-of-the-plane) component. We further assumed that the near-field amplitude *s*(*ω*, **r**) depends only on the local value of *ε*_*α*_(*ω*, **r**). We adopted the standard Lorentzian model for the hBN permittivity,

1 $$\frac{{\varepsilon _\alpha \left( \omega \right)}}{{\varepsilon _{\infty ,\alpha }}} = 1 + \frac{{\omega _{{\mathrm{LO}},\alpha }^2 - \omega _{{\mathrm{TO}},\alpha }^2}}{{\omega _{{\mathrm{TO}},\alpha }^2 - \omega ^2 - i\omega {\mathrm{\Gamma }}_\alpha }},$$

where *ω*_TO,*α*_ (*ω*_LO,*α*_) is the transverse (longitudinal) optical phonon frequency and Γ_*α*_ is the broadening^35^, which we treated as adjustable parameters. We numerically simulated the near-field scattering amplitude *s*(*ω*) modeling the tip as an elongated conducting spheroid and taking into account the presence of the quartz substrate underneath hBN^10^. Through these simulations we found that the nano-IR contrast can be attributed to the broadening of the phonon resonance, from Γ_⊥_ = 6.5 cm^−1^ at the domain centers to 7.35 cm^−1^ at the walls. The dashed green trace in Fig. 3f illustrates the effect of this extra broadening alone. In addition, a minute blue shift of *ω*_TO,⊥_ from 1365.5 to 1365.8 cm^−1^ helps to better account for the spectral form of *s*_d_(*ω*)/*s*_sol_(*ω*), as shown by the red line in Fig. 3f (for more details of these fits, see Supplementary Information Section 4).

Our analysis of the nano-IR line-form in Fig. 3 is phenomenological. The microscopic approach can be developed by relating the phonon frequency shift Δ*ω*_TO,⊥_ to the local strain. Notably, the frequency shift caused by the strain is not a single number. As shown by recent first-principles calculations^26^, a uniaxial strain splits the degenerate TO mode of hBN into two separate modes of orthogonal in-plane polarization. Moreover, from symmetry consideration we can predict that for an arbitrary strain, the fractional frequency shifts of these two modes should be:

2 $$\frac{{{\mathrm{\Delta }}\omega _{{\mathrm{TO}}, \bot }}}{{\omega _{{\mathrm{TO}}, \bot }}} = - \frac{{A + B}}{2}\left( {u_{xx} + u_{yy}} \right) \pm \sqrt {\left[ {\frac{{A - B}}{2}\left( {u_{xx} - u_{yy}} \right)} \right]^2 + \left( {Cu_{xy}} \right)^2}$$

where *A*, *B*, *C* ~ 1are constants (Supplementary Information Section 5). Effectively, the strain turns hBN from the uniaxial hyperbolic material into a bi-axial one, analogous to molybdenum trioxide^37,38^. Because of different polarization, the two modes have different coupling to the in-plane field, which complicates the modeling. Even more arduous task is to take into account the realistic three-dimensional strain distribution around the solitons. The dependence of the strain on the in-plane coordinates is relatively weak because the characteristic width *w* ~ 90 nm of the strained region near the surface is large (Figs. 1 and S2). Nevertheless, the dependence of the strain on the depth *z* is strong because of the anisotropic character of the strain (Fig. 3a and Supplementary Information Section 2). Leaving a quantitative study for future work, we limit ourselves to the following estimate. Qualitatively, the nano-IR amplitude measured above the soliton can be viewed as the sum of signals from all the underlying hBN layers, each with a shifted (and split) *ω*_TO,⊥_. Assuming the characteristic strain variation of *δu* ~ *b*/*w* ~ 0.3%, the nano-IR line-shape is expected to acquire an additional inhomogeneous broadening ΔΓ_⊥_ ~ *ω*_TO,⊥_*δu* ~ 4cm^−1^. This estimate is of the same order of magnitude as the fit parameter quoted above.

## Discussion

The confinement of both the topographic and nano-IR contrast to narrow regions along the domain walls is consistent with the notion that the individual layers in hBN are not rigid but instead behave as deformable atomic membranes prone to incommensurate–commensurate transitions. The key signature of these transitions is the formation of structural solitons where mechanical strain is accumulated^11–15^. Previously, the transmission electron microscopy and second harmonic generation experiments have identified isolated solitons^39^ in few-layer-thin hBN specimens. Our work demonstrates that the solitons can form in the interior of a bulk vdW crystal, not just at the surface or in few-layer systems, and that they can organize themselves in large regular superlattices. We have also demonstrated that nano-IR imaging can be applied to map the local strain field in a polar crystal^26,36^.

The observed residual strain concentrated in the solitons, could originate from multiple sources, one of which is the difference in the thermal expansion coefficients of hBN and graphene deposited on top of it (−8 × 10^−6^/K for graphene^40^ and −3 × 10^−6^/K for hBN^41^). A rough estimate of the lattice mismatch that could develop during the cooling of the sample is [(−8)–(−3)] **×** 10^−6^/K×(1150–20) K = 0.6%, which is close to the critical mismatch for the commensurate–incommensurate transition in an hBN bilayer^42^.

Finally, we mention several predictions testable by atomic-resolution or stacking-sensitive probes. Unlike TBG, where the lowest-energy stacking is AB stacking and the domains are triangular^1,6,14,18^, the hexagonal domains in hBN are of AA′ type (Figs. 2a and 3e), the lowest-energy stacking in hBN^43^. For the domain-wall junctions in hBN, we expect two distinct stacking types, AB1′ and AB2′, that alternate in space (Fig. 2a). Future studies may also seek to observe changes in the electronic structure in hBN moiré superlattices^44^. More broadly, it will be fruitful to explore *in operando* tuning of the twist angle^45^ and attendant lattice, electronic^2,46^, plasmonic^15^ and possibly excitonic^8,9^ responses of various vdW materials.

## Methods

### Sample synthesis

The hBN flakes were prepared on quartz substrates using mechanical exfoliation. hBN/Quartz samples were then transferred into chemical vapor deposition chamber and annealed at 1150 °C at low pressure with continuous argon flow of 50 standard cubic centimeters per minute (s.c.c.m.) for 30 min. After that, CH_4_:H_2_ at 5:5 s.c.c.m. were injected into the chamber for 300 min at pressures below 12 mbar—a process used for graphene growth^39^. Finally, samples are cooled to room temperature in argon flow.

### Nano-IR imaging

The IR nano-imaging experiments were performed using s-SNOM (neaspec.com) equipped with continuous wave mid-IR quantum cascade lasers (daylightsolutions.com). The s-SNOM is based on AFM with curvature radius ~ 25 nm operating in the tapping mode with a tapping frequency around 270 kHz. A pseudo-heterodyne interferometric detection module was implemented to extract both the scattering amplitude *s* and the phase of the near-field signal. In the current work, we discuss the amplitude of the signal. In order to subtract the background signal, we demodulated the near-field signal at the third harmonics of the tapping frequency. All the IR nano-imaging experiments were performed in ambient conditions. We used quantum cascade lasers with tunable frequency and a broad-band difference frequency generation laser systems.

## Supplementary information

Supplementary Information

## Data Availability

The data supporting the findings of this work are available from the corresponding author upon reasonable request.
