# Supplementary material for: Soliton superlattices in twisted hexagonal boron nitride
Source: Nat Commun. 2019 Sep 25;10:4360. doi: 10.1038/s41467-019-12327-x (PMC6761185; doi:10.1038/s41467-019-12327-x)
Supplement: Supplementary file 1 — Supplementary Information [file 41467_2019_12327_MOESM1_ESM.pdf]

Supplementary Information

Soliton superlattices in Twisted Hexagonal Boron Nitride

G. X. Ni et al.

1. Raman spectroscopy

In SFig. 1, we plot a typical Raman spectrum for our hBN specimens. The full width at half maximum (FWHM) of the in-plane (E_{2g}) phonon mode is $8.5\ \mathrm{cm}^{-1}$, attesting to the high quality of our samples. Note that this FWHM presumably includes some inhomogeneous broadening due to the soliton network, similar to the TO (E_{2u}) phonon mode we studied by the s-SNOM.

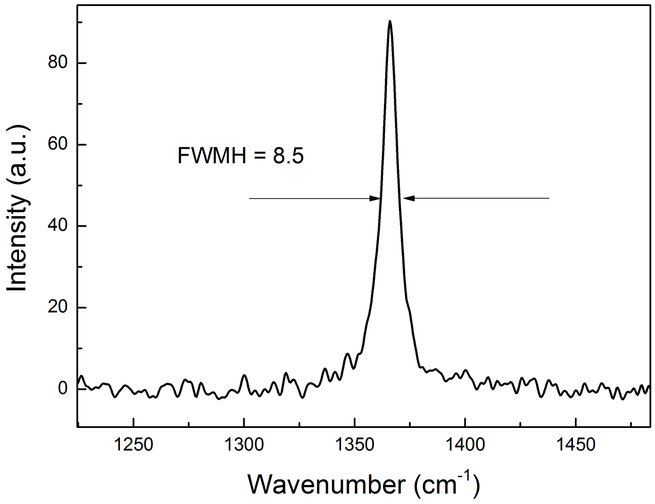

Supplementary Figure 1 | Raman spectrum of the hBN crystal.

2. Local strain and the dislocation depth estimate

Misfit dislocation networks have been studied extensively. Hexagonal networks resembling those that we observed in hBN generically appear at twist grain boundaries [S1]; References [S2, S3, S4] offer examples of experimental images for various materials. Such networks are also common in epitaxial films [S5, S6]. If the network period is large, the dislocations are far apart, and so in the first approximation we can consider each of them separately. The analysis is greatly simplified by the fact that hBN belongs to the class of hexagonal crystals, for which exact solutions for the strain distribution around single dislocations are available. Consider the case of a screw dislocation, which corresponds to a "shear" soliton [S7]. (For edge or mixed-type dislocations, the equations are more involved.) In an infinite crystal, a screw dislocation positioned on the y-axis produces the in-plane strain [S1]

$$u_{xy}^{\infty}(x,z) = \frac{b}{4\pi}\frac{\xi z}{x^2+\xi^2 z^2}\,, \qquad \xi = \sqrt{C_{66}/C_{44}}\,. \tag{S1}$$

Equation S1 implies that the strain is concentrated in two regions shaped as elliptic cylinders, which are located above and below the dislocation line (Fig. 3a of the main text). We find the anisotropy parameter $\xi \sim 7$ using the hBN elastic constants $C_{66} = (C_{11} - C_{12})/2 = 320$ GPa and $C_{44} \sim 7$ GPa from the literature [S8, S9]. Since $\xi \gg 1$, the strain has a much wider spread along x (in-plane) than along z (out-of-plane). This is a consequence of the weak interlayer coupling in hBN. In a crystal of finite thickness, the strain produced by a dislocation buried at depth d below the top surface ($z = 0$), can be found using the method of images, $u_{xy}(x, z) = u_{xy}^{\infty}(x, z + d) + u_{xy}^{\infty}(x, z - d) + \cdots$. Here we showed explicitly only the two terms that dominate the strain distribution near the top surface. The characteristic width w of the strained region at the surface is $w = \xi d$. Assuming it corresponds to the width ~ 90 nm of the minima seen in the AFM topography (SFig. 2, SFig. 3 & SFig. 4), we get the estimate of the dislocations depth $d = w/\xi \sim 15$ nm given in the main text. A more refined modeling of the strain (including buckling effects [S12, S13]) is a challenging task we leave for future work.

We observed different superlattice patterns in a large variety of hBN microcrystals (at least dozen). Therefore, the effects reported in our work are highly reproducible. In SFig. 2, 3&4 we display additional representative examples of both AFM topography and nano-IR images obtained at different frequencies. The relatively large height variations in SFig. 3 could originate from several factors (Refs. [S10, S11]). For example, collective multilayer effects, such as buckling are known to produce large high variations (Refs. [S12, S13]). It is also possible that the AFM does not measure the true height because the deformable nature of thin hBN flakes. However, we would like to emphasize that the height determination in SFig. 3 does not enter into inferences of our strain map studies. In our strain analysis, we focused on the planar distortions, which captures the key experimental results. Hence, given the imperfections of the height determination, our conclusions are still valid.

We also observed of wrinkles and folds in hBN microcrystals. Wrinkling and folding structures are a product of a high temperature (>1000 ºC) annealing process. The wrinkles are likely to form due to the mismatch of the thermal expansion coefficient between the hBN (-3 x 10^{-6} /K) and the underlying quartz substrate (5.5 x 10^{-7} /K). Consequently, a local strain would be expected near the wrinkling structures in hBN [ref. 37].

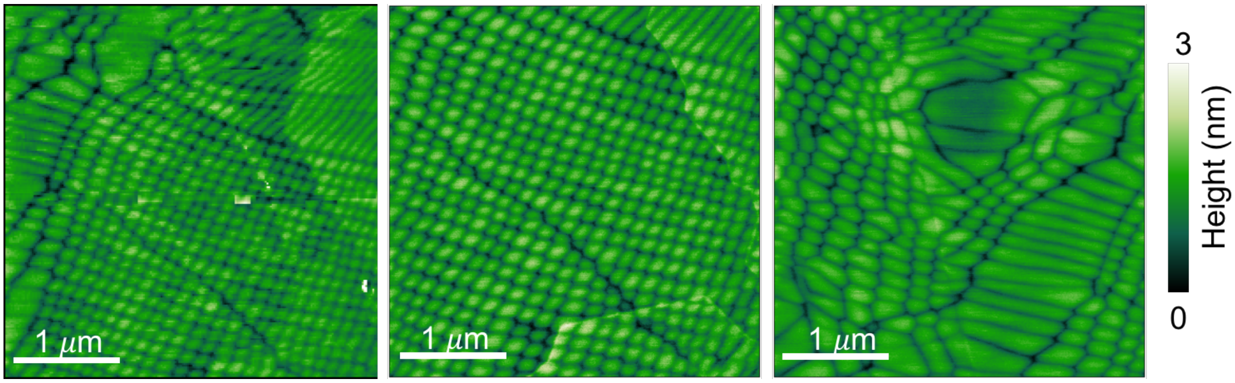

Supplementary Figure 2 | AFM topography images of hBN crystals with different pattern structures.

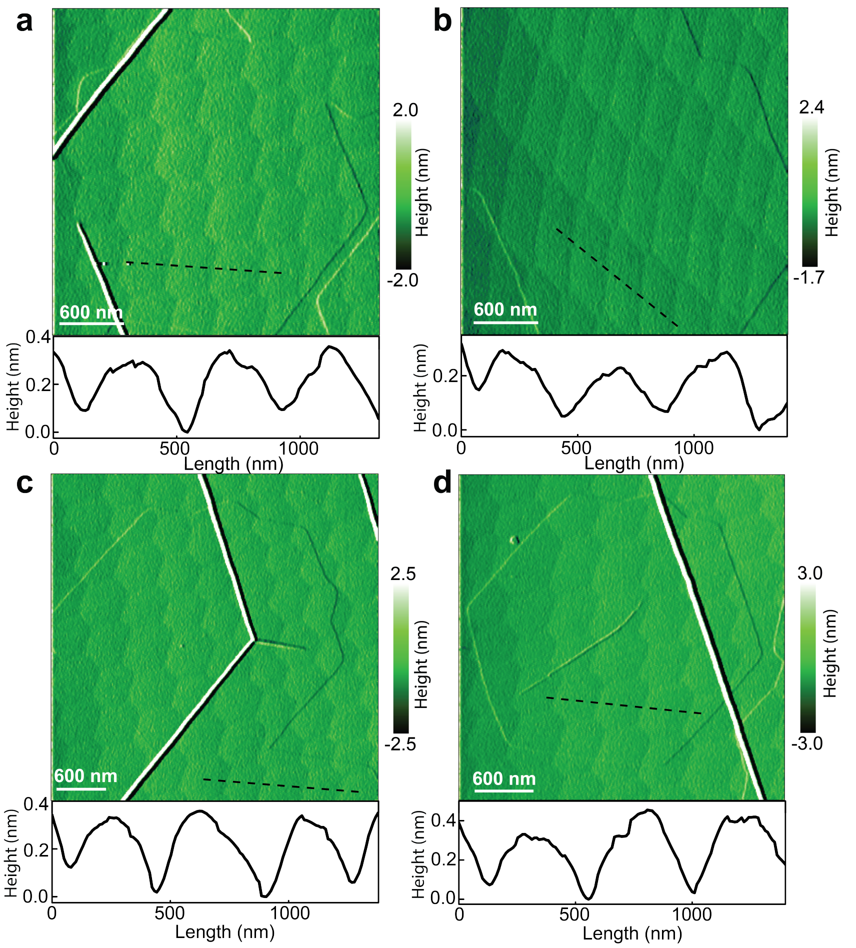

Supplementary Figure 3 | AFM topography profiles. Panels **a-d** are topography images and the corresponding line-cuts across the images shown in, respectively.

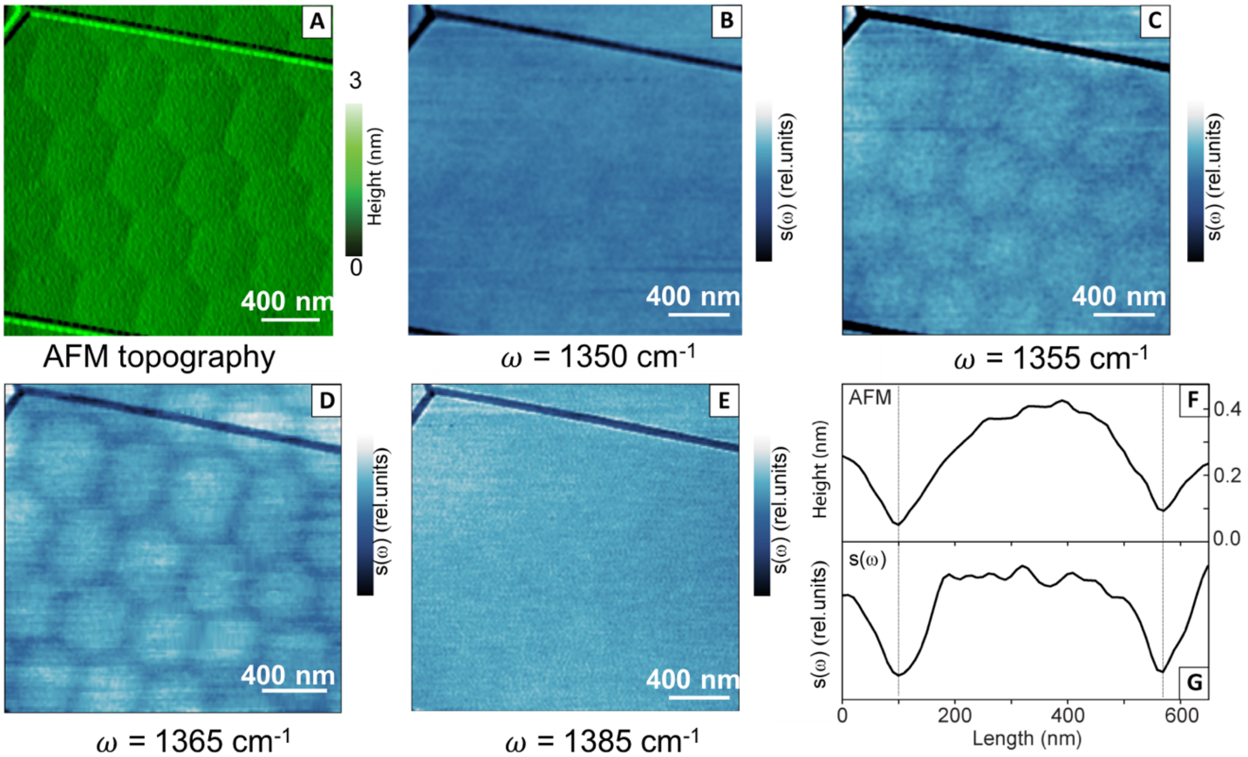

Supplementary Figure 4 | Representative nano-IR images of soliton superlattices in hBN. Panel A: Topography image. Panel B-E: nano-IR images at selected frequencies. These images were taken at the same as area as Panel A. Panel F: Topography line-profile across a moiré unit cell; Panel G: Phonon contrast across the same moiré unit cell as Panel F.

3. Coarse-grained strain and the Plateau law of dislocation networks

The coarse-grained in-plane strain tensor $\bar{u}_{\alpha\beta}$ and the twist angle $\bar{\theta}$ of the domain pattern (Fig. 2 of the main text) have been calculated according to the formulas

$$\bar{u}_{\alpha\beta} = \frac{1}{4\pi}\sum_{j=1}^{2} b_{j,\alpha}G_{j,\beta} + b_{j,\beta}G_{j,\alpha}\,, \qquad \bar{\theta} = \frac{1}{4\pi}\sum_{j=1}^{2}\left(\mathbf{G}_j \times \mathbf{b}_j\right)_z\,, \tag{S2}$$

where $\mathbf{G}_j$ is the reciprocal vector conjugate to the real-space period $\mathbf{\Lambda}_j$. These equations have been derived by noticing that whenever the observation point is shifted by either of the two primitive periods, $\mathbf{r} \to \mathbf{r} + \mathbf{\Lambda}_j$, the interlayer displacement gets incremented by the Burgers vector $\mathbf{b}_j$ of a corresponding soliton (SFig. 5a). Given the domain shape, domain size, and the orientation of the crystal lattice that defines the directions of the Burgers vectors $\mathbf{b}_j$, the calculation of $\bar{u}_{\alpha\beta}$ and $\bar{\theta}$ is straightforward. Conversely, explaining why the observed domains have such shapes and sizes presents a theoretical problem. Below we show that the location of the soliton junction $\mathbf{R}$ within the unit cell of the network is fixed by the simple rule: the arms of each junction are separated by $120°$ angles. This rule resembles the Plateau law (PL) obeyed by

foam films [S14]. Note that due to the simple parallelogram geometry of the unit cell, this position $\mathbf{R}_{\mathrm{PL}}$ is unique and independent of the Burgers vectors $\mathbf{b}_j$ unknown in our experiment.

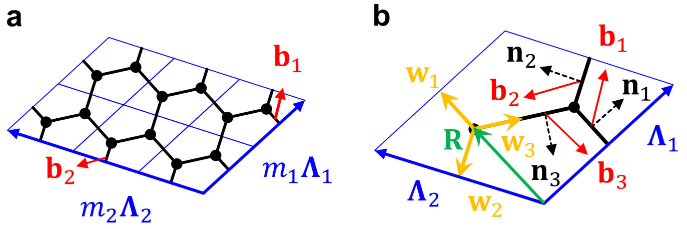

Supplementary Figure 5 | Vectors determining the domain shape (a) Domain wall configuration over many unit cells. (b) Within a unit cell, the domain wall directions $\mathbf{w}_j$ are characterized by the Burgers vectors $\mathbf{b}_j$, the normal vectors $\mathbf{n}_j$, and the location $\mathbf{R}$ of the domain-wall junction.

The PL can be derived as follows. The coarse-grained strain that causes the formation of the network originates from a misfit atomic plane boundary in the crystal. This misfit can be considered an external strain imposed on the system. We define the corresponding external stress $\sigma_{\alpha\beta} = C_{\alpha\beta\gamma\delta}\bar{u}_{\gamma\delta}$ where $C_{\alpha\beta\gamma\delta}$ is the (in-plane part) of the elastic constants tensor. If the system is in equilibrium, the size and shape of the solitons must provide the lowest total energy of the system under such stress. Neglecting the soliton-soliton interactions and the curvature of the soliton lines, the energy of each soliton has two parts. One part is the self-energy $E_{\mathrm{sol}} = \gamma(\phi)|\mathbf{w}|$, where $|\mathbf{w}|$ is the length of the soliton and γ is the line tension, i.e., the soliton energy per unit length. The latter in general depends on the angle ϕ between the Burgers vector $\mathbf{b}$ and the normal vector $\mathbf{n} = \hat{\mathbf{w}}\times\hat{\mathbf{z}}$ of the wall. The other part of the energy,

$$E_{\mathrm{ext}} = -|\mathbf{w}|\sigma_{\alpha\beta}b_\alpha n_\beta = -\sigma_{\alpha\beta}b_\alpha(\mathbf{w}\times\hat{\mathbf{z}})_\beta\,, \qquad \text{(S3)}$$

represents coupling of the soliton to the external stress. To find the position $\mathbf{R}$ of the junction, we need to minimize the total energy per unit cell,

$$E_{\mathrm{tot}} = 2\sum_{j=1}^{3}(E_{\mathrm{sol}}+E_{\mathrm{ext}})\,, \qquad \text{(S4)}$$

where the factor of two appears because there are two junctions in each cell. A virtual displacement of $\mathbf{R}$ by $\Delta\mathbf{R}$ modifies all three $\mathbf{w}_j$'s by the same amount $\Delta\mathbf{w} = -\Delta\mathbf{R}$. The corresponding change to E_{ext} vanishes,

$$\Delta E_{\mathrm{ext}} = (\Delta\mathbf{R}\times\hat{\mathbf{z}})_\beta\sigma_{\alpha\beta}\sum\nolimits_j b_{j,\alpha} = 0, \qquad \text{(S5)}$$

because the Burgers vectors $\mathbf{b}_j$ add up to zero. This means that the "external energy" is independent of $\mathbf{R}$. We only need to minimize the soliton self-energy. Taking the derivative of the self-energy with respect to $\mathbf{R}$, we get, after some algebra, Eq. (19-2) of [S1]:

$$\sum_j \left[\gamma(\phi_j)\hat{\mathbf{w}}_j + (\hat{\mathbf{z}}\times\hat{\mathbf{w}}_j)\gamma'(\phi_j)\right] = 0\,. \qquad \text{(S6)}$$

For the special case $\mathbf{R} = \mathbf{R}_{\mathrm{PL}}$ where the three solitons are oriented $120°$ apart, all three ϕ_j's are equal since $\mathbf{b}_j$'s are also oriented $120°$ apart. Additionally, $\sum_j \hat{\mathbf{w}}_j = 0$, and so Eq. (S6) is satisfied. Hence, $\mathbf{R}_{\mathrm{PL}}$ is the extremum of the self-energy. We verified numerically that this extremum is indeed the global minimum. This establishes the PL. In foams [S14], the PL follows immediately from Eq. (S6) because the surface tension γ is a constant. In our system, γ depends on the soliton direction. However, the PL is saved by the three-fold rotational symmetry of the hBN atomic plane. Note that the AFM images shown in Fig. 2c-e & f-h of the main text indicate some deviations from the PL, which may be because of the approximations made in the model or because of dislocation pinning by residual defects, which prevents the system from reaching full equilibrium.

4. Fitting of the nano-IR contrast

To simulate $s_{\mathrm{d}}(\omega)/s_{\mathrm{sol}}(\omega)$ spectra, we employed the electromagnetic solver developed previously [S15, S16]. It requires as an input the infrared reflectivity of the sample, which we calculated by accounting all the layers (air/hBN/quartz) present in the system. For hBN we used the Lorentzian permittivity model, Eq. (1) of the main text. SFigure 6 shows the calculated $s_{\mathrm{d}}(\omega)/s_{\mathrm{sol}}(\omega)$ spectra for different choices of two adjustable parameters: the damping $\Gamma_{\perp}$ and the optical phonon frequency $\omega_{\mathrm{TO},\perp}$. Specifically, the $s_{\mathrm{d}}(\omega)$ spectra can be fitted with the parameters $\omega_{\mathrm{TO},\perp} = 1365.5\ \mathrm{cm}^{-1}$ and $\Gamma_{\perp} = 6.5\ \mathrm{cm}^{-1}$, which is consistent with previous work [S17, S18, S19]. The best fit to the $s_{\mathrm{sol}}(\omega)$ spectra is obtained with $\omega_{\mathrm{TO},\perp} = 1365.8\ \mathrm{cm}^{-1}$ and $\Gamma_{\perp} = 7.35\ \mathrm{cm}^{-1}$.

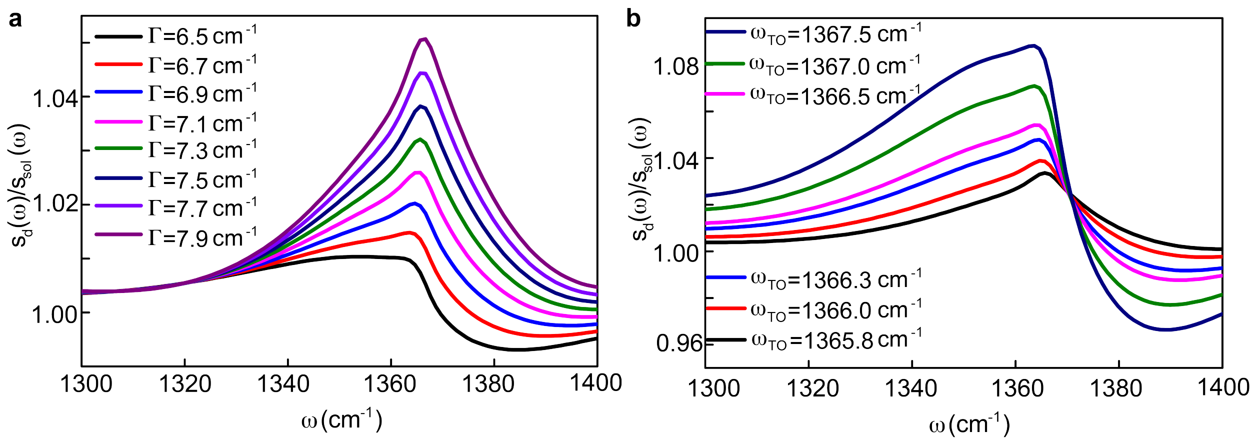

Supplementary Figure 6 | $s_{\mathrm{d}}(\omega)/s_{\mathrm{sol}}(\omega)$ spectra fitting. a: $s_{\mathrm{d}}(\omega)/s_{\mathrm{sol}}(\omega)$ spectra at different $\Gamma_{\perp}$. b: $s_{\mathrm{d}}(\omega)/s_{\mathrm{sol}}(\omega)$ spectra at different $\omega_{\mathrm{TO},\perp}$.

5. Phonon frequency shift due to strain

The frequencies of the two-fold degenerate E_{2u} phonon mode are the eigenvalues of a certain

$2{\times}2$ dynamical matrix $D_{\alpha\beta}$. The perturbative effect of a weak strain on this matrix is described by the second-order elastic tensor $G_{\alpha\beta\gamma\delta}$. If we restrict attention to the in-plane coordinates, the hexagonal symmetry of the hBN planes implies that this fourth-rank tensor has only three linearly independent nonzero elements, e.g., $A = G_{xxxx}$, $B = G_{xxyy}$, and $C = G_{xyxy}$. Therefore, the strain produces the perturbation of the form

$$\Delta D_{\alpha\beta} = G_{\alpha\beta\gamma\delta} u_{\gamma\delta} = \begin{pmatrix} Au_{xx} + Bu_{yy} & Cu_{xy} \\ Cu_{xy} & Bu_{xx} + Au_{yy} \end{pmatrix} \tag{S7}$$

Diagonalizing this matrix, we obtain Eq. (2) of the main text.

Supplementary References

S1. Hirth, J. P. and Lothe, J., Theory of dislocations (Krieger, Malabar, FL, 1982).

S2. Forwood, C. T. and Clarebrough, L. M., Hexagonal arrays of grain-boundary dislocations in coherent twin boundaries, Phil. Mag. Lett. A 53, L31-L34 (1985). DOI: https://doi.org/10.1080/01418618608242828

S3. Zhang, H., Zhang, C., Hu, T., Zhan, X., Wang, X., Zhou, Y., On the small angle twist sub-grain boundaries in Ti_3AlC_2, Sci. Rep. 6, 23943 (2016). DOI: https://doi.org/10.1038/srep23943

S4. Amelinckx, S. and Delavignette, P., Observation of dislocations in non-metallic layer structures, Nature 185, 603-604 (1960). DOI: https://doi.org/10.1038/185603a0

S5. Yamaguchi, H., Belk, J. G., Zhang, X. M., Sudijono, J. L., Fahy, M. R., Jones, T. S., Pashley, D. W., and Joyce, B. A., Atomic-scale imaging of strain relaxation via misfit dislocations in highly mismatched semiconductor heteroepitaxy: InAs/GaAs(111)A, Phys. Rev. B 55, 1337 (1997). DOI: https://doi.org/10.1103/PhysRevB.55.1337

S6. Chen, C., Wang, Z., Kato, T., Shibata, N., Taniguchi, T., and Ikuhara, Y., Misfit accommodation mechanism at the heterointerface between diamond and cubic boron nitride, Nature Comm. 6, 6327 (2015). DOI: https://doi.org/10.1038/ncomms7327

S7. Jiang, B.-Y. *et al.* Plasmon Reflections by Topological Electronic Boundaries in Bilayer Graphene, *Nano Lett*. **17**, 7080 (2017).

S8. Bosak, A., Serrano, J., Krisch, M., Watanabe, K., Taniguchi, T., and Kanda, H., Phys. Rev. B **73**, 041402(R) (2006). DOI: https://doi.org/10.1103/PhysRevB.73.041402

S9. Lebedev, A.V., Lebedeva, I.V., Knizhnik, A.A., Popov, A.M. Interlayer interaction and related properties of bilayer hexagonal boron nitride: ab initio study. *RSC Adv.* **6**, 6423 (2016).

S10. Yankowitz, M. et al. Pressure-induced commensurate stacking of graphene on boron nitride. Nature Comm. 7, 13168 (2016).

S11. Kim, K. et al. Tunable moiré bands and strong correlations in small-twist-angle bilayer graphene. PNAS, 114, 3364 (2017).

S12. Siems, R., Delavignette, P., and Amelinckx, S., The buckling of a thin plate due to the presence of an edge dislocation, Phys. Stat. Sol. (B) 2, 421-438 (1962). DOI: https://doi.org/10.1002/pssb.19620020407

S13. Butz, B., Dolle, C., Weber, F. N. D., Waldmann, D., Weber, H. B., Meyer, B., and Spiecker, E., Dislocations in bilayer graphene, Nature 505, 533 (2014). DOI: https://doi.org/10.1038/nature12780

S14. Weaire, D. and Hutzler, S. The Physics of Foams (Oxford University Press, Oxford, 1999).

S15. Fei, Z. *et al.* Infrared Nanoscopy of Dirac Plasmons at the Graphene-SiO_2 interface. *Nano Lett.* **11**, 4701-4705 (2011).

S16. Dai, S. *et al.* Tunable Phonon Polaritons in Atomically Thin van der Waals Crystals of Boron Nitride. *Science* **343**, 1125-1129, (2014).

S17. Caldwell, J. D. *et al*. Sub-diffractional, volume-confined polaritons in a natural hyperbolic material: hexagonal boron nitride. *Nature Commun*, **5**, 5221 (2014).

S18. Principi, A. *et al*. Plasmon losses due to electron-phonon scattering: the case of graphene encapsulated in hexagonal Boron Nitride. *Phys. Rev. B* **90**, 165408 (2014).

S19. Ni, G. X. et al. Fundamental limits to graphene plasmonics. *Nature* **557**, 530-533 (2018).
